# Comparative proteomics of ovaries elucidated the potential targets related to ovine prolificacy

**DOI:** 10.3389/fvets.2023.1096762

**Published:** 2023-08-22

**Authors:** Chunyan Li, Mei Zhou, Xiaoyun He, Ran Di, Zijun Zhang, Chunhuan Ren, Qiuyue Liu, Mingxing Chu

**Affiliations:** ^1^Key Laboratory of Animal Genetics, Breeding and Reproduction of Ministry of Agriculture and Rural Affairs, Institute of Animal Science, Chinese Academy of Agricultural Sciences, Beijing, China; ^2^Yunnan Provincial Engineering Laboratory of Animal Genetic Resource Conservation and Germplasm Enhancement, Yunnan Academy of Animal Husbandry and Veterinary Sciences, Kunming, China; ^3^Key Laboratory of Pig Molecular Quantitative Genetics of Anhui Academy of Agricultural Sciences, Anhui Provincial Key Laboratory of Livestock and Poultry Product Safety Engineering, Institute of Animal Husbandry and Veterinary Medicine, Anhui Academy of Agricultural Sciences, Hefei, China; ^4^College of Animal Science and Technology, Anhui Agricultural University, Hefei, China

**Keywords:** sheep, prolificacy, ovaries, tandem mass tag, protein

## Abstract

Small Tail Han (STH) sheep, a unique Chinese breed, is recognized for its early maturity, year-round estrus, and prolificacy. However, the molecular mechanism of its high prolificacy has not been fully elucidated. The Proteomics approach is feasible and effective to reveal the proteins involved in the complex physiological processes of any organism. Given this, we performed the protein expression profiling of ovarian tissues during the luteal phase using polytocous STH sheep (litter size ≥2, three consecutive lambings) and monotocous STH sheep (litter size =1, three consecutive lambings) (PL vs. ML), and the follicular phase using polytocous STH sheep (litter size ≥2, three consecutive lambings) and monotocous STH sheep (litter size =1, three consecutive lambings) (PF vs. MF), respectively. Parallel Reaction Monitoring (PRM) was conducted to validate the differentially abundant proteins (DAPs). The tandem mass tag (TMT) quantitative proteomic results showed that a total of 5,237 proteins were identified, of which 49 and 44 showed differential abundance in the PL vs. ML and PF vs. MF groups, respectively. Enrichments analyses indicated that the DAPs including TIA1 cytotoxic granule-associated RNA-binding protein-like 1 (TIAL1), nicotinamide phosphoribosyltransferase (NAMPT), and cellular retinoic acid-binding protein 1 (CRABP1) were enriched at the luteal phase, while TIAL1, inhibin beta-a-subunit (A2ICA4), and W5PG55 were enriched at the follicular phase, potentially mediating reproductive processes in polytocous ewes. Furthermore, six DAPs were verified using PRM, confirming the accuracy of the TMT data acquired in this study. Together, our work expanded the database of indigenous sheep breeds and provided new ovarian candidate molecular targets, which will help in the study of the genetic mechanisms of ovine prolificacy.

## Introduction

Litter size is an important reproduction indicator in sheep breeding; an increase in litter size has an effective impact on the profitability of the mutton industry, promoting the supply of meat, milk, and wool for daily life in modern agriculture. However, litter size has low heritability (approximately 0.1) (1), which is influenced by many factors including ovarian growth, estrus, ovulation rate, and uterine receptivity (2, 3). The multiple molecular mechanisms for litter size have not yet been fully elucidated. Most studies of genetic mechanisms underlying the prolificacy in sheep so far have focused on candidate genes and their regulators such as lncRNA, circRNA, and multiple regulated pathways, which are probably interrelated, to facilitate ovine reproductive performance (4, 5). Prolific and non-prolific ewes are characterized by differences in the estrous cycle. The genes *PRLR* (6), *BMP15* (7, 8), *GDF9* (9, 10), *FecB* (11, 12), *BMPR1B* (13), *NTRK2* (14), and *LHβ* (15), as well as crucial molecular pathways like BMP/Smad (16, 17) and TGF-beta signaling pathways (18) are involved in follicular development and increased ovulation rate and are proven to be closely associated with the prolificacy trait of sheep. Additionally, intraovarian peptides and growth factors also participate in the regulation of ovarian activity through paracrine or autocrine pathways, thus ovarian activity, in turn, determines the estrus cycle (19).

Proteomics is a feasible and effective biotechnology that can provide global protein information including structure, abundance, function, localization, interaction, and modification. Advancement in the proteomic technique has enhanced the sensitivity and accuracy of the identification and quantification of proteins or peptides in a cell, tissue, or organism. The recently developed tandem mass tag (TMT) labeling combined with mass spectrometry (MS) has been used as a particularly robust proteomic approach in different kinds of research fields, detecting quantitative protein information in multiple biological samples (20). To date, TMT reagents have been executed to analyze protein biomarkers in plasma (21) to assess differentially expressed proteins between follicle-stimulating hormone (FSH)-positive and -negative non-functional pituitary adenoma in patients (22) and to identify potential biomarkers in sub-clinical mastitis in cows (23). Also, we previously demonstrated a few potential proteins involved in ovine litter size traits such as STAR, HSD3B1, and CYP11A1 using the TMT-based technique (24–26). Moreover, parallel reaction monitoring (PRM) has emerged as a powerful targeted proteomic approach that has been verified for protein quantification because of its higher sensitivity, resolution, accuracy, and reproducibility (27).

Small Tail Han (STH) is a remarkable polytocous maternal ovine breed in northern China known for its year-round estrus and high prolificacy, particularly with the lambing rate coming up to 267.1% (26). This breed has become an excellent prolific model for the research of ovine high fecundity. In this study, a TMT-based quantitative proteomic technique was applied to map proteome profiling of the ovaries in STH ewes with two fecundity traits in order to characterize the proteins’ abundance and search for candidate proteins related to fecundity. Furthermore, the PRM assay was also performed to verify the TMT results. The identification of important proteins combined with the relevant bioinformatics analysis will replenish potential biomarkers in revealing the underlying mechanism of prolificacy in sheep.

## Materials and methods

### Ethics statement

All experimental procedures were performed following the relevant guideline and regulations set by the Ministry of Agriculture of the People′s Republic of China. Ethical approval on animal survival was given by the animal ethics committee of the Institute of Animal Science, Chinese Academy of Agricultural Sciences (No. IAS 2019-49).

### Experimental design and workflow

TMT technology was used to identify and characterize the differentially abundant proteins (DAPs) in the ovaries of polytocous and monotocous ewes at the luteal and follicular phases, respectively. Each group of biological samples consisted of ovarian tissues isolated from three STH sheep. The protein extraction, TMT labeling, and mass spectrometry were performed for protein identification, followed by bioinformatics analysis (Supplementary Figure S1).

### Animal grouping and sample collection

Experimental STH ewes were selected from the Chenglian STH sheep breeding farm in the southwest region of Shandong Province, P. R. China. They were housed under the same condition, with free access to water and food. Firstly, jugular vein blood of healthy non-pregnant ewes (*n* = 890) was collected which was used for the identification of the *FecB* genotypes using TaqMan assay (28), thus three genotypes were obtained. The ewes chosen were approximately aged 2–4 years and weighed 65–75 kg. A total of 12 ewes (six polytocous with *FecB^BB^* genotype and six monotocous with *FecB*^++^ genotype) with similar age and weight indexes were then selected for our study. Estrus synchronization was performed on the whole selected ewes, where a controlled internal drug-releasing device (CIDR, progesterone 300 mg, Zoetis Australia Pty. Ltd., NSW, Australia) was put into these ewes′ vagina for 12 days, and 5 mL of vitamin AD was intramuscularly injected to protect the vaginal epithelium. Ovulation rate (the number of corpus luteum) was detected by the laparoscopy procedure after 7 days from the CIDR removal (luteal phase), which is a key index determining sheep prolificacy. Then, the estrus synchronization mentioned above was performed again after 45 h from the CIDR removal, and ovaries with follicle diameter ≥ 3.5 mm were obtained (follicular phase) (12, 29). The 12 ewes were divided into four groups, including polytocous ewes in the follicular phase (PF, *n* = 3), polytocous ewes in the luteal phase (PL, *n* = 3), monotocous ewes in the follicular phase (MF, *n* = 3), and monotocous ewes in the luteal phase (ML, *n* = 3). Fresh entire ovarian tissues from 12 ewes were collected, frozen immediately in liquid nitrogen, and stored at −80°C for subsequent proteomic experiments.

### Protein extraction, digestion, and TMT labeling

Firstly, according to the method given by Tang et al. (25), the protein was extracted by lysis with SDT lysate (including 4% w/v SDS, 150 mM Tris/HCl (pH8), and 100 mM DTT). Also, 30 mg of each ovarian tissue was added to 900 μL SDT; the lysate was sonicated (80 W, 10 cycles of 10 s with 15 s intervals) and then boiled for 15 min. After centrifugation at 14,000 × g for 40 min, the final supernatant was quantified using a BCA assay (Bio-Rad, CA, United States) and detected by SDS-PAGE (Supplementary Figure S2). Protein was treated with the filter-aided sample preparation (FASP) method for enzymatic hydrolysis (30), and the filtrate was collected. The peptide fraction was qualified at 280 nm, OD280. After trypsin digestion, the peptide samples were dried by vacuum centrifugation, then 100 μg peptides of each sample were resuspended in 0.5 M TEAB. The TMT labeling kit (Thermo Fisher Scientific, Waltham, MA, United States) with the isobaric labels 126, 127, 128, and 129 was used to label the PF, PL, MF, and ML samples, respectively.

### Peptide fractionation with high pH reversed-phase spin column

The peptide mixture was fractionated using the pierce high pH reversed-phase fractionation kit (Thermo Fisher Scientific, Waltham, MA, United States) according to the manufacturer′s instructions.

### LC–MS/MS analysis

The freeze-dried samples were dissolved in solvent A (0.1% formic acid, v/v) and loaded onto an Acclaim PepMap 100 pre-column (reversed-phase C18, Thermo Fisher) at a flow rate of 300 nL/min and then separated on an Acclaim pepMap RSLC analytical column (reversed-phase C18, Thermo Fisher). The gradient was comprised of an increase in solvent B (84% acetonitrile with 0.1% formic acid) as follows: 0–35% for 50 min, 35–100% for 5 min, and 100% for 5 min. LC–MS/MS analysis was performed on a Q Exactive mass spectrometer that was coupled to an Easy-nLC 1,000 UPLC system (Thermo Fisher Scientific, Waltham, MA, United States) for 60 min. Positive ions were detected, and the parameters of the precursor ions scan ranged from 300 to 1,800 m/z. Primary MS resolution was 70,000 at 200 m/z, automatic gain control (AGC) was 3e6, maximum inject time was 10 ms, and dynamic exclusion duration was 40.0 s. Secondary MS resolution was 17,500 at 200 m/z, maximum inject time was 1.60 ms, and normalized collision energy was 30 eV, with an underfill ratio of 0.1%.

### MS database searching and DAPs screening

The raw MS data were output in RAW format and searched using the database (uniprot_Ovis_aries_27500_20170904.fasta), which was converted to the mascot generic format (MGF) file format using Proteome Discoverer 1.4 (Thermo Fisher Scientific, 2012) and retrieved through the Mascot 2.2 server^1^ to identify and quantify proteins. The related parameters were as follows: maximum missed cleavages = 2; fixed modifications = Carbamidomethyl (C), TMT-6 plex (N-term), and TMT-6 plex (K); variable modifications = Oxidation (M) and TMT-4 plex (Y); peptide ion mass tolerance = ± 20 ppm; fragment ion mass tolerance = 0.1 Da; database = uniprot_Ovis_aries_27500_20170904.fasta, database pattern = Decoy; and peptide false discovery rate (FDR) ≤0.01 as the cutoff to obtain highly reliable qualitative results. The protein ratios were calculated as the median of only unique peptides of the protein, all peptide ratios were normalized by the median protein ratio, and the median protein ratio should be one after the normalization. The *p*-values of trusted proteins calculated with Student′s *t*-test showed significant differences (fold-change >1.2 or < 0.833; adjusted *p*-value <0.05) between groups.

### Parallel reaction monitoring verification

In order to validate the proteomic data obtained by TMT analysis, the PRM method (a technique based on MS analysis) was used to determine the abundance levels of selected ovarian function-related DAPs (unique peptides ≥2, fold change (FC) >1.2 or < 0.833) (31). After enzymatic hydrolysis, the peptides were desalted, lyophilized, and redissolved in 0.1% formic acid. The concentration of the peptides was determined by OD280. The peptides’ information suitable for the PRM analysis was imported into the Xcalibur™ software (Thermo Fisher Scientific) for the PRM setting. Each sample contained 1 μg peptides, and 200 fmol standard peptides were added (PRTC: GILFVGSGVSGGEEGAR) for chromatographic separation using the HPLC system. Q-Exactive HF MS (Thermo Fisher Scientific) was used for the PRM/MS analysis for 60 min and positive ions were detected. All PRM scans were performed according to the inclusion list after each first-order full mass spectrum scan. The raw data of LC-PRM/MS were analyzed using Skyline software (MacCoss Lab, University of Washington) (32).

### Bioinformatics analysis

GO enrichment^2^ analysis for identifying protein was performed using Blast2GO software, BLAST tool^3^ as the protein sequence database, mapping, annotation, and annotation augmentation. KAAS software was used to annotate the KEGG pathway,^4^ mapping DAPs to the KEGG genes database; the mapped proteins were categorized based on KEGG orthology. GO and KEGG pathways were executed using Fisher′s exact test (20). Additionally, protein–protein interaction (PPI) networks of DAPs were performed by STRING^5^ combined with Cytoscape software to attempt to capture the underlying protein interaction information.

## Results

### Analysis of MS data

**Figure 1:**
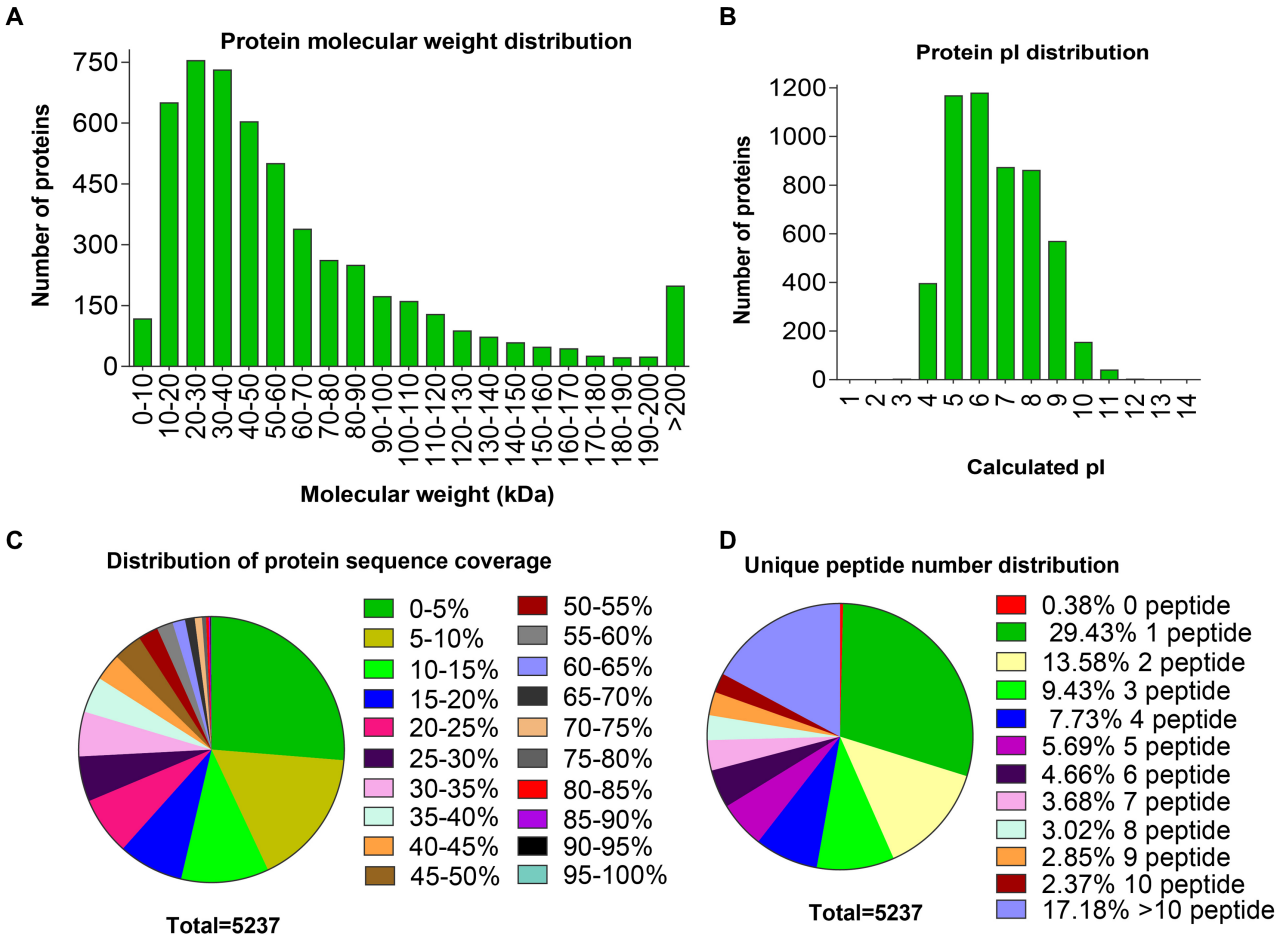
Distributions of molecular weight, pI, sequence coverage, and number of unique peptides of proteins identified in the ovaries. **(A)** Distribution of protein molecular weights. **(B)** Distribution of protein pI values. **(C)** Distribution of protein sequence coverage. **(D)** Distribution of unique peptide numbers.

Ovarian tissues from two comparison groups were isolated and pooled for protein extraction, TMT labeling, and MS analysis. A total of 167,020 peptide spectrum matching numbers and 4,749 unique peptides were obtained in this study (Supplementary Table S1), which were mapped to 5,237 proteins, wherein 732 were uncharacterized and 4,505 were known functions. A complete list of the proteins and peptides detected in the ovary, with the UniProt accession number, protein name, and the score number of unique peptides and amino acids, molecular weight as well as sequence coverage are shown in Supplementary Table S2. Among these results, most distributions of the protein molecular weights (MWs) ranged from 10 to 200 kDa (Figure 1A), and the isoelectric point (pI) values ranged from 4 to 10 (Figure 1B). More than half the protein sequence coverage was ≤15% (Figure 1C). Most identified proteins contained more than one or even up to 201 unique peptides, wherein 29.4% of the proteins had only one unique peptide (Figure 1D).

### Identification of differentially abundant proteins

**Figure 2:**
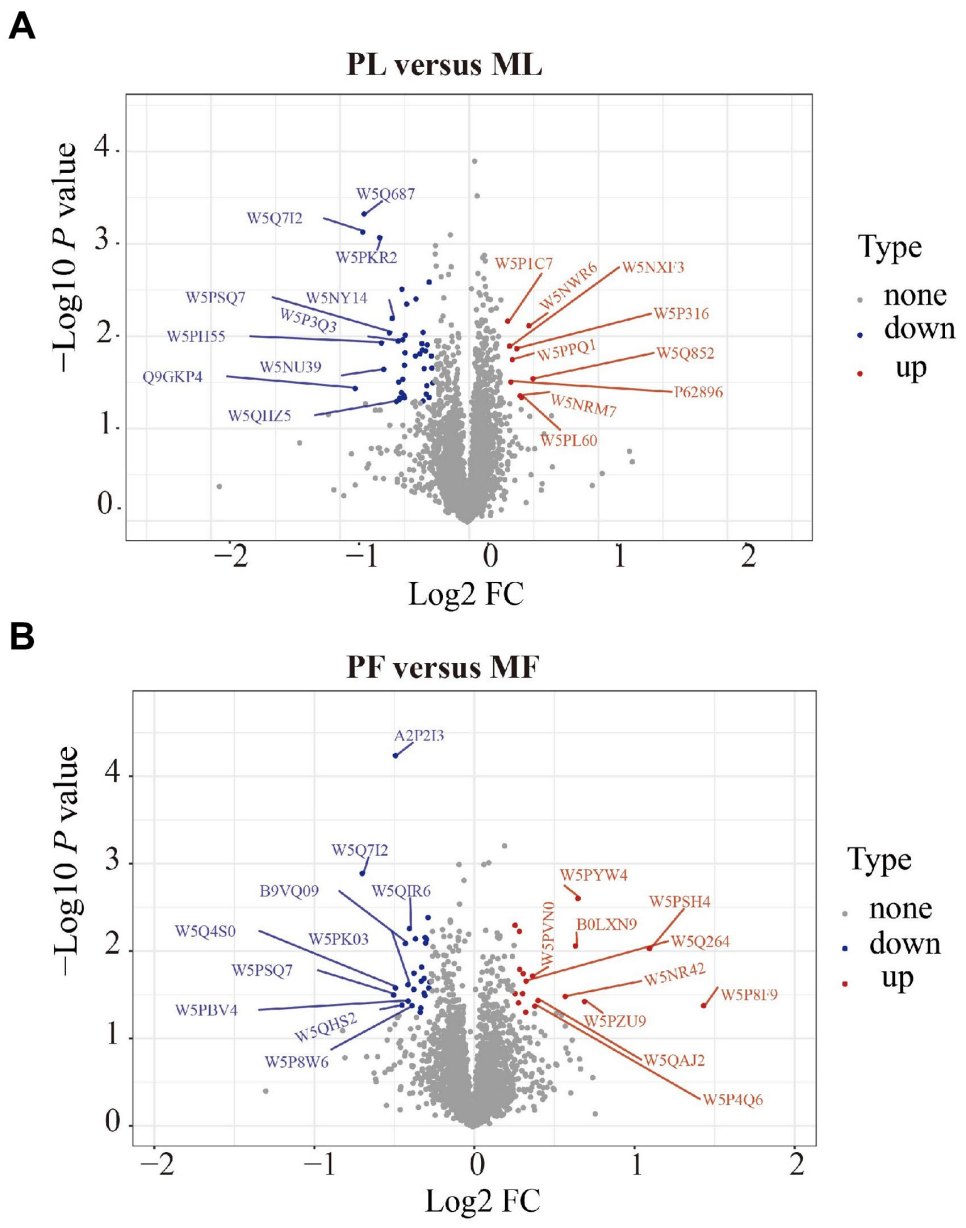
Volcano plots of DAPs in PL vs. ML **(A)**, and in PF vs. MF **(B)**. Where the red circles on the positive side represent upregulated proteins and those on the negative side represent downregulated proteins.

**Figure 3:**
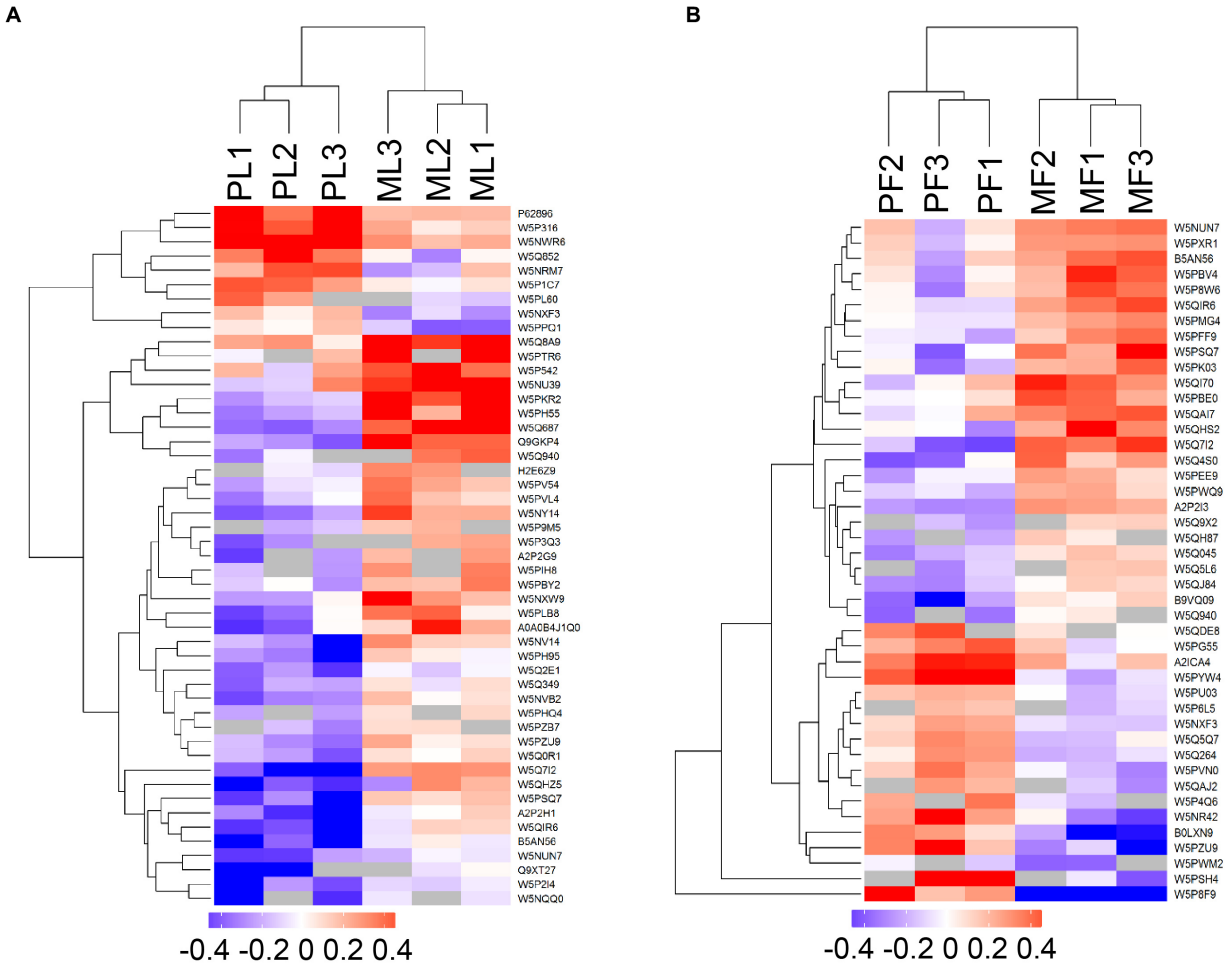
Heatmaps of DAPs in PL vs. ML **(A)**, and PF vs. MF **(B)**. Where the red and blue shades represent significantly upregulated and downregulated proteins, respectively, and grey areas indicate no quantitative information about proteins.

DAPs among the two comparison groups (PL vs. ML and PF vs. MF) were classified according to proteins with FC > 1.20 and *p* < 0.05. There were 49 DAPs (9 upregulated and 40 downregulated) and 44 DAPs (18 upregulated and 26 downregulated) in PL vs. ML and PF vs. MF comparison groups, respectively (Figure 2B; Supplementary Table S3). In the luteal phase, the differentially abundant proteins, such as TIA1 cytotoxic granule associated RNA binding protein-like 1 (TIAL1), nicotinamide phosphoribosyl transferase (NAMPT), and cellular retinoic acid-binding protein 1 (CRABP1) were higher in PL than that in ML, whereas complement component 4 binding protein alpha (C4BPA), interferon-stimulated gene 17 (ISG17), yes-associated protein 1 (YAP1), sex hormone binding globulin (SHBG), and uncharacterized protein (W5P9M5) were lower in PL than in ML. In the follicular phase, the differentially abundant proteins, including TIAL1, uncharacterized protein (W5PG55), inhibin beta-A-subunit (A2ICA4), and crystallin mu (CRYM) were higher in PF than in MF, whereas lysosomal associated membrane protein 1 (LAMP1), collagen type XII alpha 1 chain (COL12A1), and CD74 molecule (CD74) were lower in PF than in MF. Hierarchical clustering analysis of DAPs is shown in Figure 3; the similarity of protein abundance patterns within each group was relatively high, whereas it was relatively low between polytocous and monotocous groups, and the groups were clearly divided, which suggested the rationality of DAPs in this experiment.

### Functional enrichment analysis of DAPs

**Figure 4:**
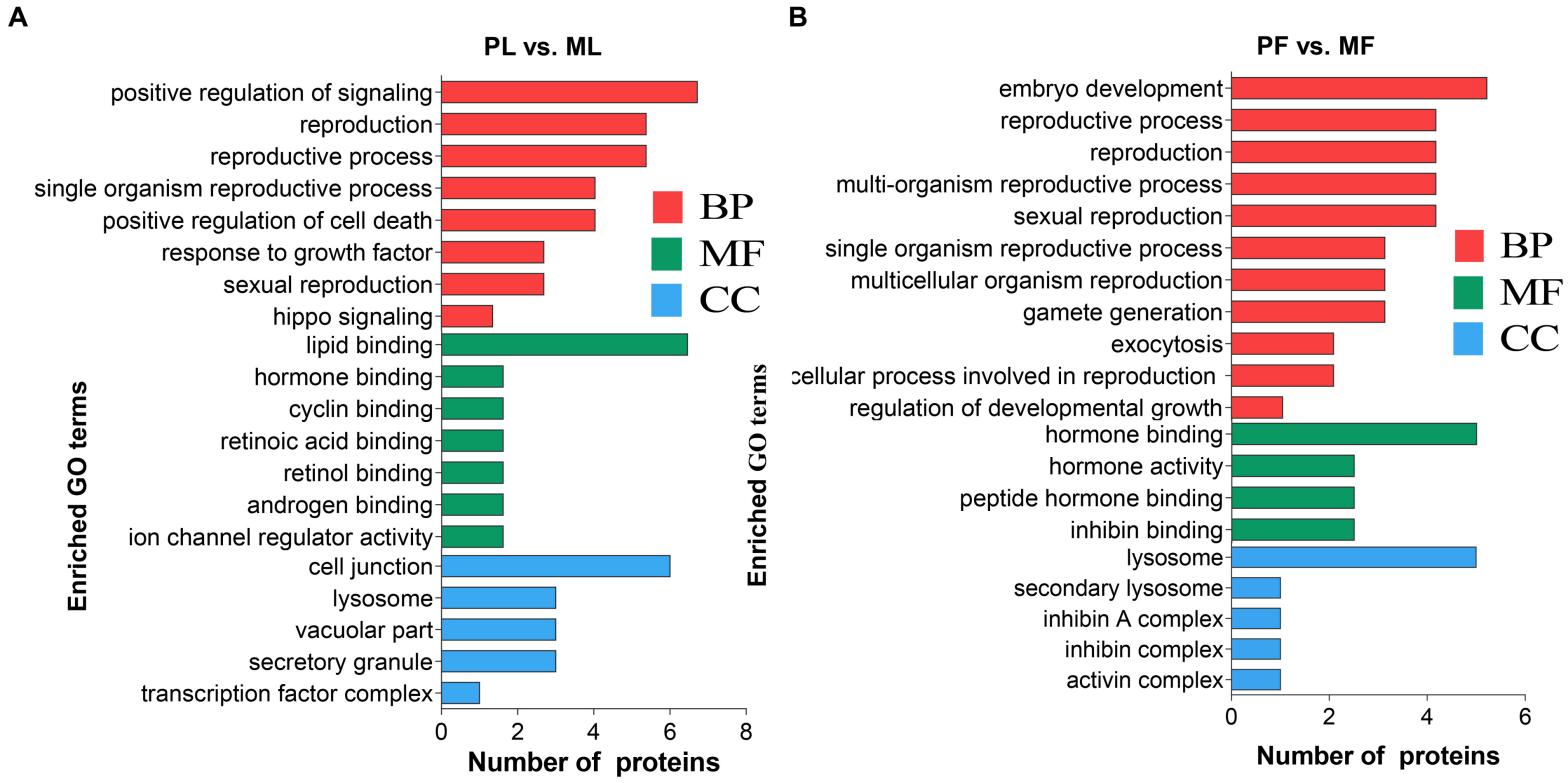
The 20 enriched GO terms related to reproduction concluding biological process (BP), molecular function (MF), and cellular component (CC) in PL vs. ML **(A)**, and PF vs. MF **(B)**.

To identify the potential roles of DAPs in the two comparison groups, enrichment analyses of GO terms and KEGG pathways were performed. In general, GO analysis contains three significant ontologies of biological functions: biological process (BP), cellular component (CC), and molecular function (MF). Among them, each DAP was assigned more than one term; the 20 enriched GO terms related to reproduction are shown in Figure 4. In the PL vs. ML group, the BP terms included positive regulation of signaling, reproductive process, and sexual reproduction (Figure 4A; Supplementary Table S4), as well as predominantly enriched proteins including TIAL1, NAMPT, CRABP1, C4BPA, ISG17, W5P9M5, and SHBG proteins, while gamete generation, embryo development, reproductive process, and cellular process involved in reproduction in multicellular organisms were enriched in PF vs. MF (Figure 4B), including proteins of TIAL1, W5PG55, LAMP1, and A2ICA4.

**Figure 5:**
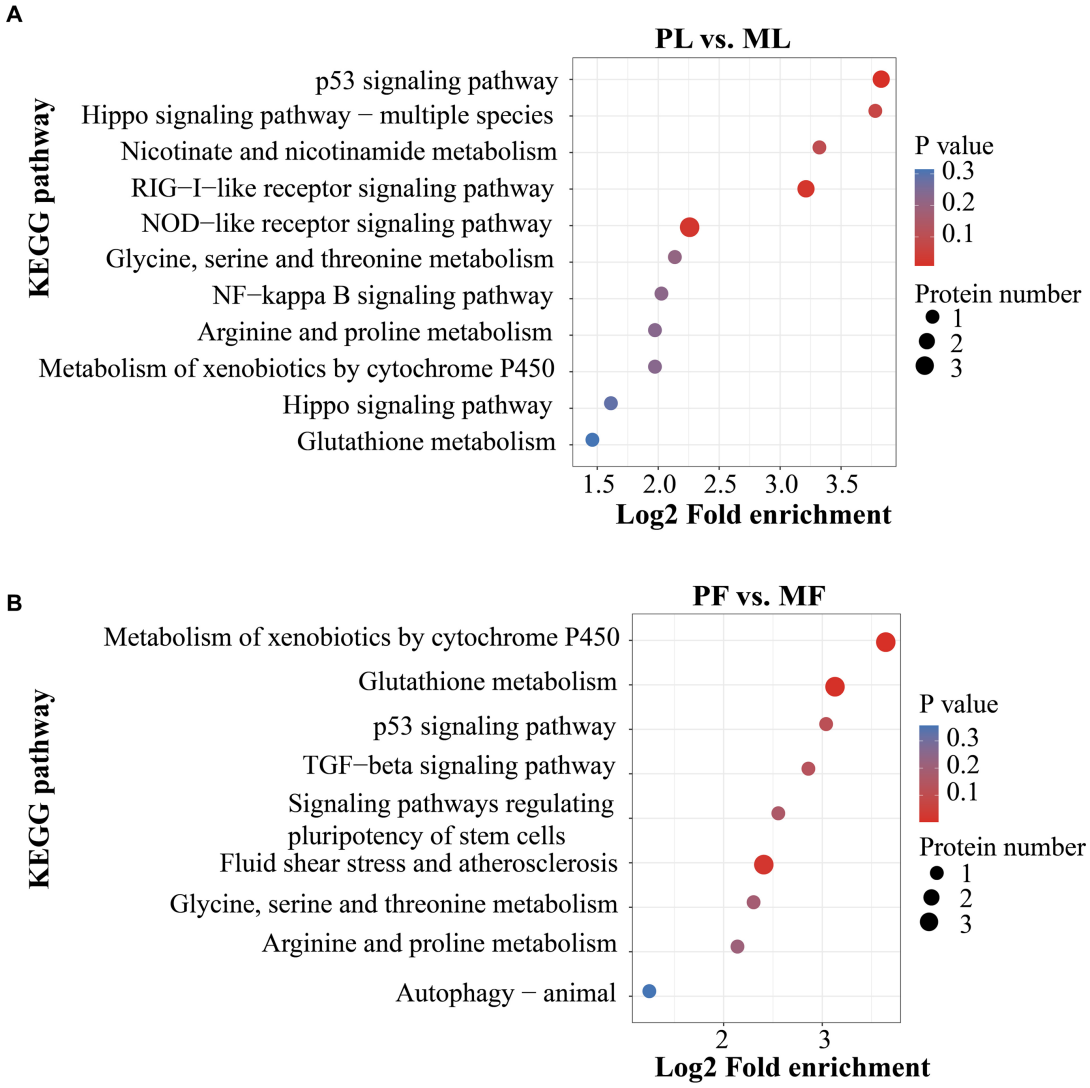
KEGG pathway analyses of DAPs in PL vs. ML **(A)**, and in PF vs. MF **(B)**.

In addition, DAPs in PL vs. ML and PF vs. MF comparison groups were enriched in 64 and 42 pathways, respectively (Supplementary Table S5). *p* < 0.05 was set as the cutoff for further enrichment analysis. In the luteal phase, several pathways related to ovarian function were significantly enriched such as nicotinate and nicotinamide metabolism (NAMPT), hippo signaling (W5P9M5), NOD-like receptor signaling (NAMPT, W5NY14, and W5PH55), and RIG-I-like receptor signaling pathways (ISG17 and DDX58) (Figure 5A). In the follicular phase, pathways related to follicular development were significantly enriched, including TGF-beta signaling and signaling pathways regulating the pluripotency of stem cells (A2ICA4) (Figure 5B). In general, GO and KEGG enrichment analyses provided crucial clues on the molecular mechanisms underlying ewes′ prolificacy.

### PPI network analysis of DAPs

**Figure 6:**
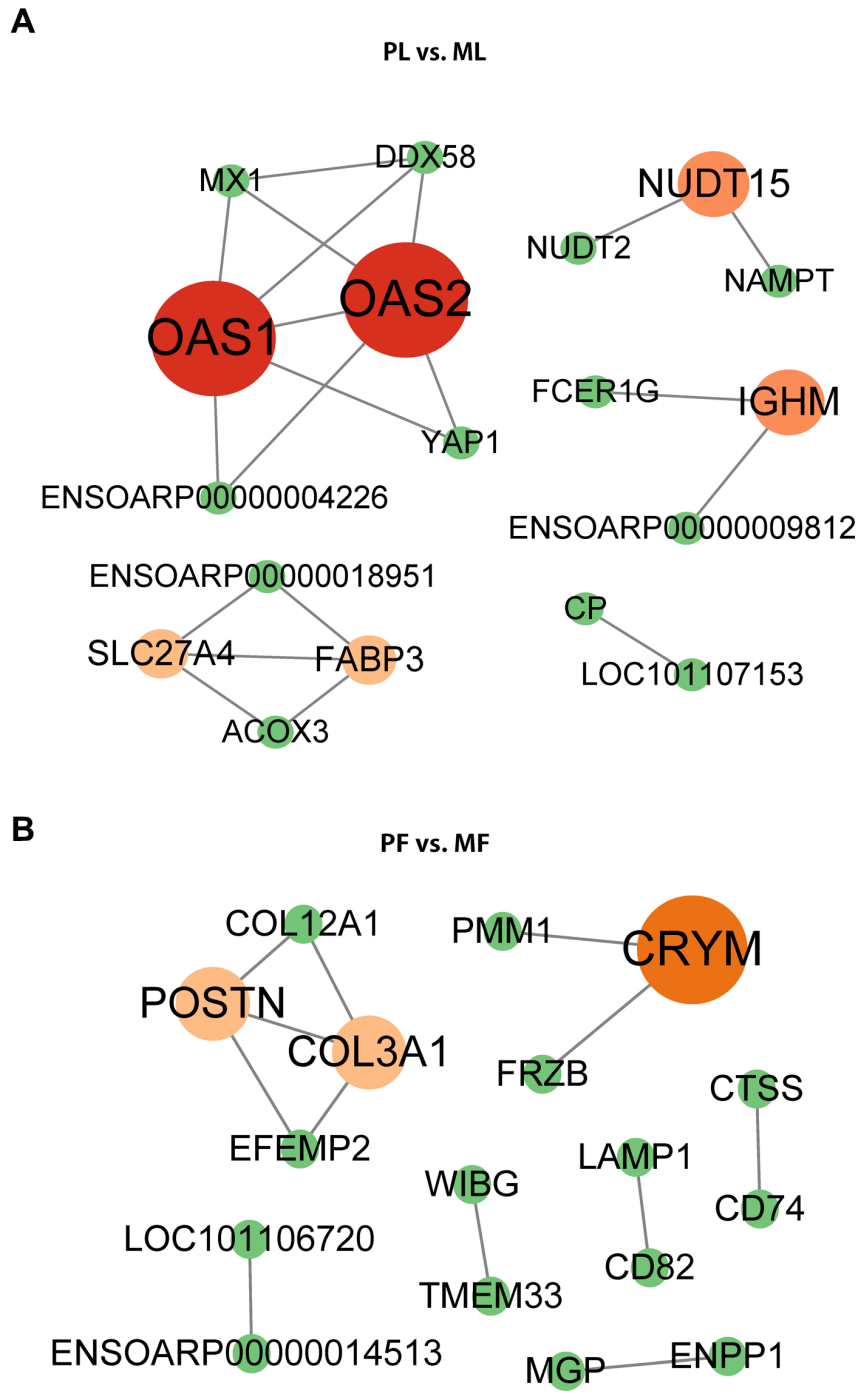
PPI network of DAPs in PL vs. ML **(A)**, and PF vs. MF **(B)**. Different colors represent the betweenness score values of proteins in the process of analysis using the cystoscope (v.3.9.1).

Using the STRING database and Cytoscape software (v.3.9.1), we constructed a PPI network for the DAPs. STRING data is shown in Supplementary Table S6, and the betweenness was selected during CytoNCA analysis. Our results showed that a few DAPs were involved in the PPI, and hub proteins were found. Importantly, YAP1 and NAMPT interacted with other DAPs in PL vs. ML (Figure 6A), while COL12A1, LAMP1, and CD74 interacted with other DAPs in PF vs. MF (Figure 6B), respectively.

### PRM validation of TMT-based results

**Figure 7:**
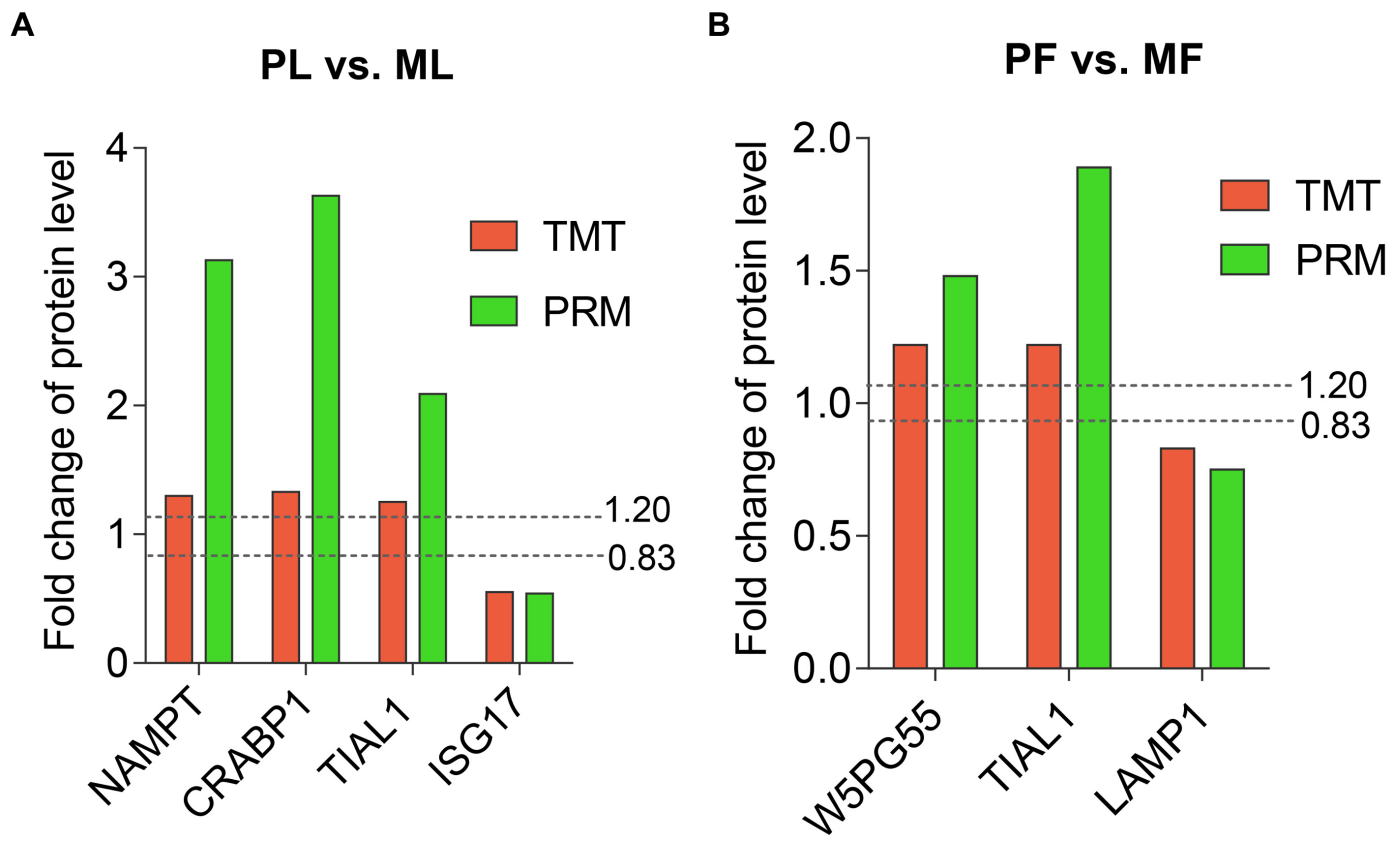
Abundant patterns of six DAPs using TMT analyses and PRM validation in PL vs. ML **(A)**, and in PF vs. MF **(B)**. Two dotted lines represent FC = 1.2 (upregulation) and FC = 0.833 (downregulation).

Six DAPs (NAMPT, CRABP1, TIAL1, and ISG17 in PL vs. ML, and W5PG55, TIAL1, and LAMP1 in PF vs. MF) containing at least two unique peptides were selected for verification by PRM quantitative analysis. Among the DAPs, FC of NAMPT, CRABP1, TIAL1, and W5PG55 were above 1.2, while ISG17 and LAMP1 were below 0.833. The abundance trend of PRM was consistent with that of TMT-based results, which indicated the credibility of our proteomics data (Figure 7; Supplementary Table S7).

## Discussion

Reproduction is an extraordinarily complex process with coordinated regulation at multiple reproductive organs and molecular levels. In particular, the precise molecular mechanism of sheep reproduction is not fully clarified. As one of the direct reproductive tissues, the ovary supports the development of follicles and oocytes (33). Investigation of this tissue from sheep in different estrus phases could provide potential information on further improvement to litter size (34). In this study, high-throughput sequencing of the proteome in ovaries involved in the prolificacy traits of STH sheep was performed in different estrus phases. Acquisition of the ovarian DAPs between polytocous and monotocous groups might participate in the molecular regulation of sheep prolificacy.

In the comparison of PL vs. ML, functional enrichment analysis of important differential proteins revealed that nicotinamide phosphoribosyl transferase (NAMPT), interferon-stimulated gene 17 (ISG17), and W5P9M5 were associated with the prolificacy trait. As a cytokine hormone and rate-limiting enzyme, NAMPT is implicated in mediating energy metabolism, apoptosis, and biosynthesis of cellular nicotinamide adenine dinucleotide (NAD)^+^, which is a crucial coenzyme for dehydrogenases in cellular metabolism (35). In a mammal, NAMPT usually cooperates with SIRT1 to regulate intracellular NAD^+^ levels and, therefore, makes available cellular energy, which is important for placental cell survival and a successful pregnancy (36). Besides, NAMPT increases insulin-like growth factor 1-induced steroidogenesis in primary granulosa cells of the human ovary (37) and improves oocyte quality and fertility during rat superovulation (38). ISG17 protein in the ovine uterus responds to interferon-tau (IFNtau), which is a major signal for maternal recognition of pregnancy (MRP). MRP might facilitate the transition from cyclicity to pregnancy (39, 40). A high abundance of ISG17 in the ovary of monotocous ewes suggested that the ISG17 may regulate uterus function with IFNtau as a downstream negative effector. In addition, we found that W5P9M5 was encoded by the *YAP1* gene and significantly enriched in the hippo signaling pathway. Research has shown that the hippo pathway is conserved and involved in spatio-temporal correlation with the size of the primordial follicle pool (41), as well as controlling ovarian follicular growth and oocyte maturation (42). YAP1 is one of the components of the hippo pathway, and its overexpression can enhance polycystic ovarian syndrome (PCOS), which is an etiology of oligo-ovulation.

Previous reports have proved that TIA1 cytotoxic granule-associated RNA binding protein-like 1 (TIAL1) was expressed in primordial germ cells and plays a role in the development of spermatogonia or oogonia (43), as well as a stress granule marker to respond to stress conditions such as porcine reproductive and respiratory syndrome virus infection, guarding reproduction of sow and health of the young (44). Our study revealed that TIAL1 was highly abundant in ovaries of polytocous ewes and associated with cytoplasmic stress granules and the regulation of cellular processes. Cellular retinoic acid-binding protein 1 (CRABP1) could directly inhibit endogenous Ca^2+^/calmodulin-dependent protein kinase II (CaMKII) by competing with calmodulin (45). While CaMKII is a key regulator of egg activation events such as completion of meiosis and progression to embryonic interphase, its activity contributes to the membrane block to prevent fertilization by polyspermy (46). The upregulation of CRABP1 in polytocous ewes further implied its inhibitory effect on CaMKII. Additionally, enrichment of A2ICA4 in the PF ovaries was mainly involved in the signaling pathway regulating the pluripotency of stem cells and the positive regulation of follicle-stimulating hormone secretion. A2ICA4 also stimulates the production of SMAD2/SMAD3, which is the downstream SMAD signaling of TGF-β1 (47) and participates in gonadal development, embryonic differentiation, and placenta formation. Lysosomal-associated membrane protein 1 (LAMP1) is expressed in reproductive tissues such as the ovaries and uterus in humans and is involved in lysosomal stability and autophagy (48). More abundance and functional enrichment of LAMP1 in MF ovaries of sheep further confirmed this protein′s role in mediating reproductive cell′s survival response such as autophagy during the follicular phase. Intriguingly, our study also revealed that W5PG55 was associated with the anatomic structure development, growth, and reproduction during the follicular phase, which is encoded by the *SELENOP* gene. The SELENOP bears an N-terminal selenocysteine-containing thioredoxin functional domain, which is indicative of its potential redox function (49). Research has shown that important proteins controlling the oxidative condition in the follicular fluid can regulate follicle growth and oocyte maturation (50). These suggest the potential antioxidative effect of the extracellular region or secreted protein W5PG55.

## Conclusion

In general, changes in protein components from ovine ovaries in two comparison groups were investigated using TMT-based quantitative proteomics, and DAPs were further characterized by bioinformatics analysis. Abundance levels of proteins in the ovary are associated with its physiological state. Totally, 49 and 44 DAPs in PL vs. ML and PF vs. MF were identified, respectively. Upregulation of TIAL1, NAMPT, and CRABP1 in PL vs. ML, and TIAL1, A2ICA4, and W5PG55 in PF vs. MF might positively modulate reproduction in polytocous ewes. These proteins are of substantial significance for the in-depth study of the genetic mechanism of ovine prolificacy.

## Data availability statement

The datasets presented in this study can be found in online repositories. The names of the repository/repositories and accession number(s) can be found in the article/Supplementary material.

## Ethics statement

The animal study was reviewed and approved by the Science Research Department (in charge of animal welfare issues) of the Institute of Animal Science, Chinese Academy of Agricultural Sciences (IAS-CAAS).

## Author contributions

CL analyzed the data, wrote, and revised the manuscript. MZ designed and performed the experiment. XH and RD prepared the samples. ZZ and CR investigated the resources. QL designed the experiment and reviewed the manuscript. MC administrated the project and revised the final manuscript. All authors contributed to the article and approved the submitted version.

## Funding

This work was supported by the National Natural Science Foundation of China (32172704), the Agricultural Science and Technology Innovation Program of China (CAAS-ZDRW202106 and ASTIP-IAS13), the Earmarked Fund for China Agriculture Research System (CARS-38), and the Central Public-interest Scientific Institution Basal Research Fund (Y2017JC24).

## Conflict of interest

The authors declare that the research was conducted in the absence of any commercial or financial relationships that could be construed as a potential conflict of interest.

The reviewer YaL declared a shared affiliation with the authors ZZ and CR to the handling editor at the time of review.

## Publisher’s note

All claims expressed in this article are solely those of the authors and do not necessarily represent those of their affiliated organizations, or those of the publisher, the editors and the reviewers. Any product that may be evaluated in this article, or claim that may be made by its manufacturer, is not guaranteed or endorsed by the publisher.

## Footnotes

^1^ http://www.matrixscience.com/

^2^ http://www.ebi.ac.uk/interpro/

^3^ https://blast.ncbi.nlm.nih.gov/

^4^ http://www.genome.jp/kaas-bin/kaasmain

^5^ https://cn.string-db.org/
