# Supplementary figures and images for: Comparative proteomics of ovaries elucidated the potential targets related to ovine prolificacy
Source: Front Vet Sci. 2023 Aug 22;10:1096762. doi: 10.3389/fvets.2023.1096762 (PMC10477366; doi:10.3389/fvets.2023.1096762)

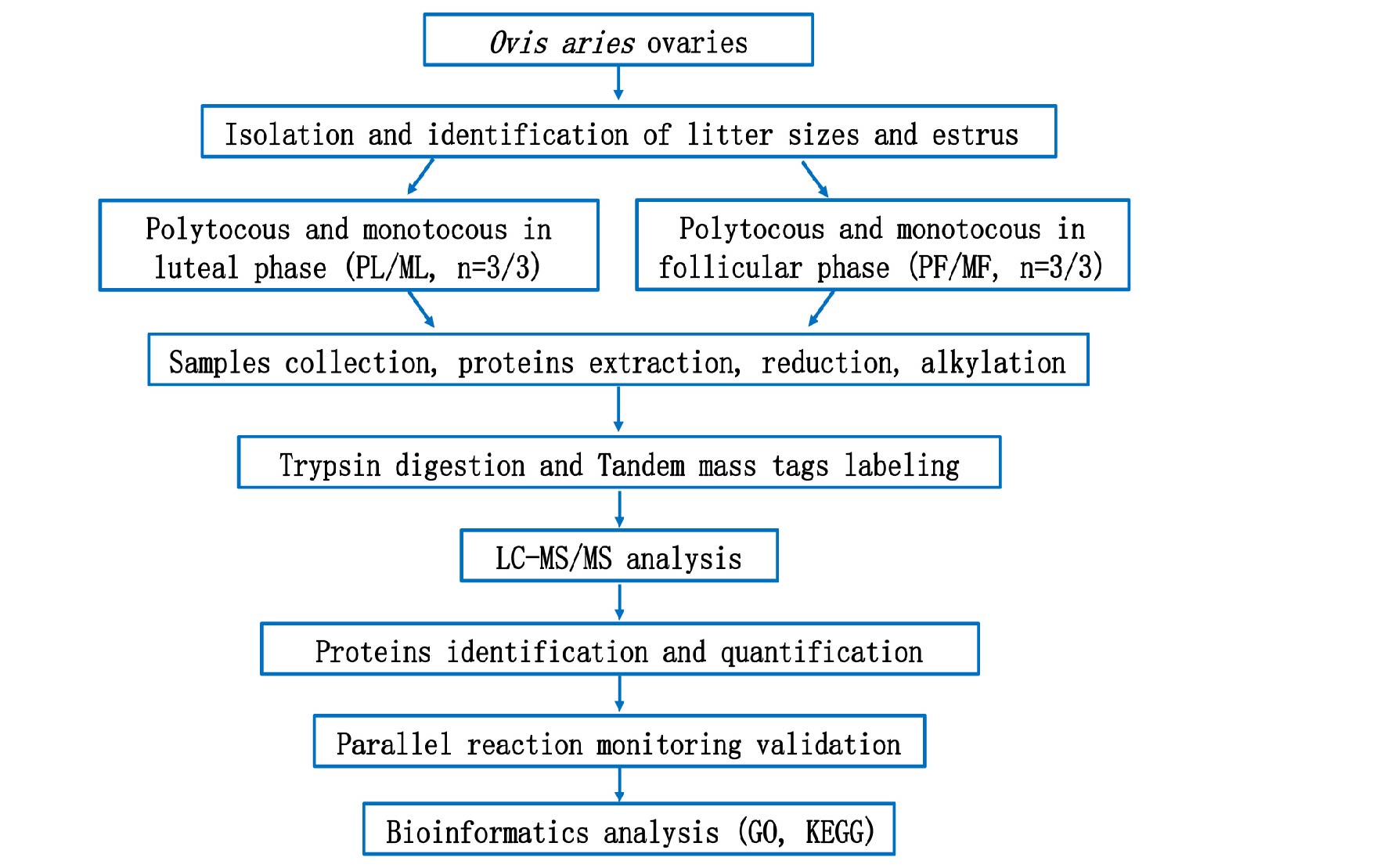

Supplement: Supplementary file 8 [file Image_1.JPEG]

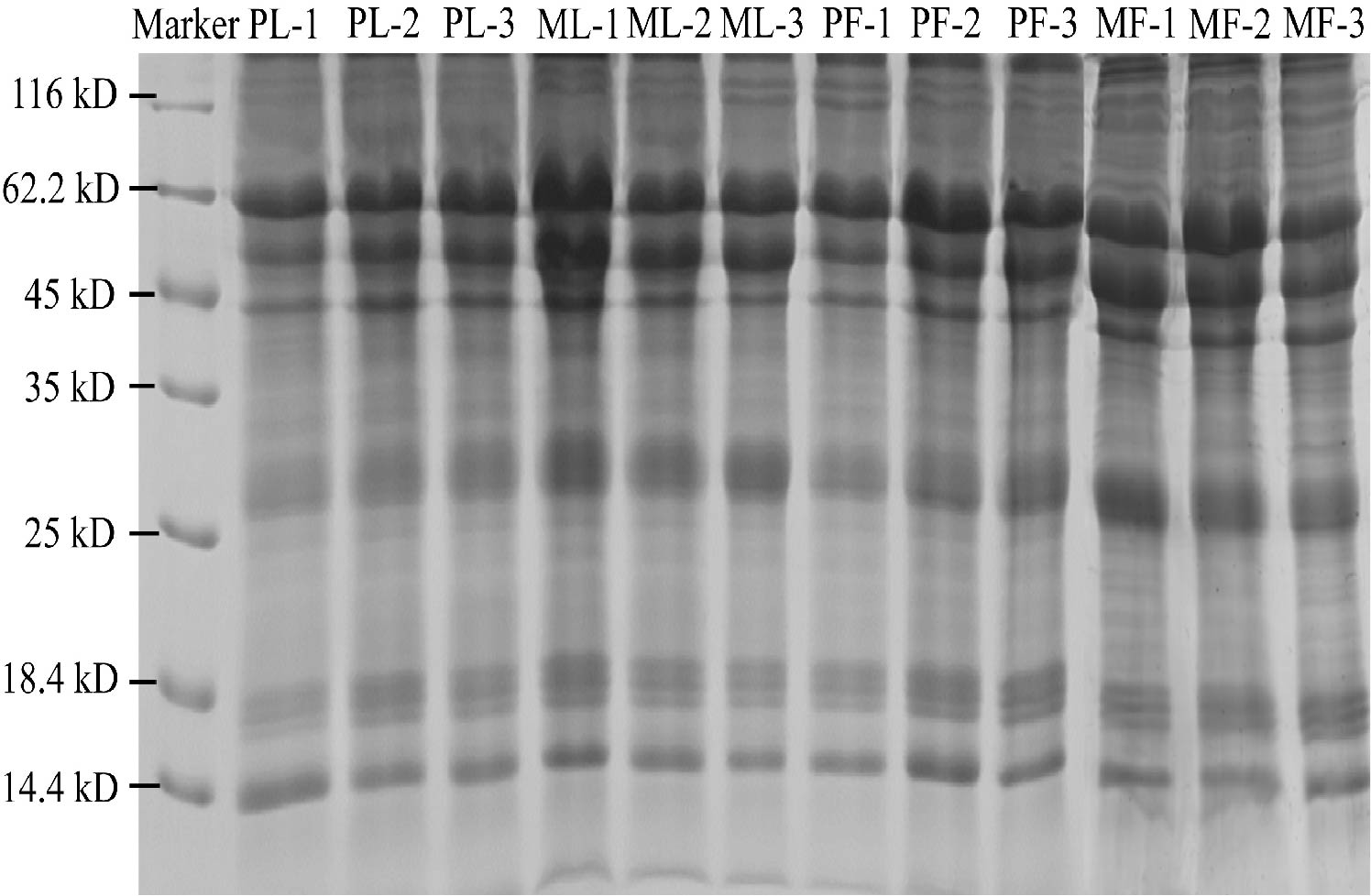

Supplement: Supplementary file 9 [file Image_2.JPEG]
